# RNA interference modulates replication of dengue virus in *Drosophila melanogaster *cells

**DOI:** 10.1186/1471-2180-10-127

**Published:** 2010-04-27

**Authors:** Swati Mukherjee, Kathryn A Hanley

**Affiliations:** 1 Molecular Biology Program, New Mexico State University, Las Cruces, NM 88003, USA; 2 Department of Biology, New Mexico State University, Las Cruces, NM 88003, USA

## Abstract

**Background:**

Mosquito-borne dengue virus (DENV, genus *Flavivirus*) has emerged as a major threat to global human health in recent decades, and novel strategies to contain the escalating dengue fever pandemic are urgently needed. RNA interference (RNAi) induced by exogenous small interfering RNAs (siRNAs) has shown promise for treatment of flavivirus infections in hosts and prevention of transmission by vectors. However, the impact of RNAi triggered by authentic virus infection on replication of DENV, or any flavivirus, has received little study. The objectives of the current study were threefold: first, to assess the utility of *Drosophila melanogaster *S2 cells for the study of DENV, second to investigate the impact of multiple enzymes in the RNAi pathway on DENV replication; and third to test for variation in the response of the four serotypes of DENV to modulation of RNAi.

**Results:**

Three strains from each of the four DENV serotypes showed replication in S2 cells following infection at multiplicity of infection (MOI) 0.1 and MOI 10; each strain achieved titers > 4.0 log_10_pfu/ml five days after infection at MOI 10. The four serotypes did not differ in mean titer. S2 cells infected with DENV-1, 2, 3 or 4 produced siRNAs, indicating that infection triggered an RNAi response. Knockdown of one of the major enzymes in the RNAi pathway, Dicer-2 (Dcr-2), resulted in a 10 to 100-fold enhancement of replication of all twelve strains of DENV in S2 cells. While serotypes did not differ in their average response to Dcr-2 knockdown, strains within serotypes showed significant differences in their sensitivity to Dcr-2 knockdown. Moreover, knockdown of three additional components of the RNAi pathway, Argonaute 2 (Ago-2), Dcr-1 and Ago-1, also resulted in a significant increase in replication of the two DENV strains tested, and the magnitude of this increase was similar to that resulting from Dcr-2 knockdown.

**Conclusions:**

These findings indicate that DENV can replicate in *Drosophila *S2 cells and that the RNAi pathway plays a role in modulating DENV replication in these cells. S2 cells offer a useful cell culture model for evaluation of the interaction between DENV and the RNAi response.

## Background

The genus *Flavivirus *contains a large number of emerging, vector-transmitted viruses. Of these, the four serotypes of dengue virus (DENV-1-4) pose the most significant threat to global public health. The global pandemic of dengue fever has escalated dramatically in recent decades, accompanied by a sharp increase in the more severe manifestations of the disease, dengue hemorrhagic fever and dengue shock syndrome [1]. Widespread cessation of vector control, increases in mosquito-breeding sites due to rapid urbanization, and expansion of global travel have all contributed to DENV emergence [2]. Vector control is a costly and often ineffective response to outbreaks [3]. No antivirals are currently available for any flavivirus [4], and although promising DENV vaccine candidates have recently entered clinical trials [5], progress in the development of a DENV vaccine has been slow [6].

**Figure 1:**
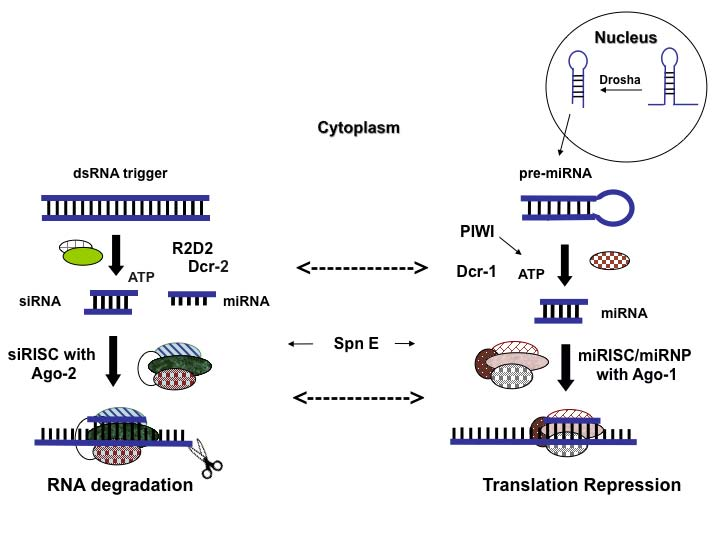
**Cartoon representing the major enzymes involved in the overlapping branches of the siRNA and the miRNA pathways in *Drosophila melanogaster***. While this cartoon was designed to emphasize the differences between the two pathways, it is important to stress that there is also extensive interaction and overlap between the two branches (some of which are represented by dotted arrows). This latter point is discussed in more detail in the text. [siRISC: RNA Induced Silencing Complex associated with siRNA; miRISC: miRNA associated RISC; miRNP: miRNA associated Ribo-Nucleo Protein complex; Ago: Argonaute (exhibits slicer activity); Dcr: Dicer; Spn-E: Spindle-E protein (involved in assembly of RISC); PIWI (co-purifies with Dcr-1 in *Drosophila *germline cells); R2D2 (bridges initiator and effector steps of siRNA pathway); ATP: adenosine triphosphate] [11,12,46,51-57].

In response to this exigency, investigators have pursued novel methods to prevent and treat dengue disease. In particular, there is considerable excitement about the potential to utilize RNA interference (RNAi) (Figure 1) to treat flavivirus infection in the host and control flavivirus transmission by the vector [7]. The RNAi pathway is composed of two major branches (Figure 1). The small interfering RNA (siRNA) branch is triggered by perfectly or nearly-perfectly base-paired exogenous dsRNA and results in RNA degradation, while the cellular microRNA branch (miRNA) is triggered by imperfectly base-paired dsRNA and results in translation repression [8-10]. Although siRNAs and miRNAs are processed via discrete pathways, specific enzymes may participate in both pathways. For example, recent evidence from *Drosophila *indicates that Dicer (Dcr)-1 is critical for both RNA degradation and translation repression, while Dcr-2 is required only for RNA degradation [11,12], and that Argonaute (Ago)-1 and Ago-2 proteins overlap in their functions [13].

Kumar et al. [14] have demonstrated that introduction of exogenous siRNAs can prevent encephalitis caused by West Nile virus (WNV) and Japanese encephalitis virus infections, and genetically-modified mosquitoes expressing siRNAs are currently being developed to prevent transmission of DENV [8,15]. However, the impact of RNAi triggered by endogenous dsRNA produced during virus infection on DENV replication, or that of any flavivirus, has received little study.

To date, only two studies have examined whether virus-triggered RNAi regulates replication of a flavivirus. Chotkowski et al. demonstrated that *Drosophila melanogaster *S2 cells infected with WNV produced abundant anti-WNV siRNAs and that knockdown of Ago-2 (Figure 1) in these cells increased the rate but not the overall level of WNV replication [16]. Moreover, *D. melanogaster *carrying homozygous null mutations in Ago-2, spindle-E (Spn-E) or PIWI (Figure 1) supported higher levels of WNV replication than wild type controls, while flies carrying homozygous null mutations in Dcr-2 (Figure 1) did not [16]. Intriguingly, *Aedes albopictus *mosquito C6/36 cells infected with WNV did not produce anti-WNV siRNA's, prompting the authors to speculate that the RNAi response in this cell line may be weaker than that of *Drosophila *cells [16,17]. However Sanchez-Vargas et al. showed that cells of *Aedes aegypti *mosquitoes, the major vector of DENV, produce anti-DENV siRNA following infection with DENV-2 in culture and *in vivo *[18]. Moreover in the latter study knockdown of Dcr-2, Ago-2, or R2D2 (Figure 1) all significantly enhanced the rate and level of DENV-2 replication, with knockdown of Dcr-2 having the strongest impact. These findings indicate that components of both the miRNA and the siRNA branches are involved in modulating viral replication, and that complete functional segregation of the two branches is lacking.

To gain further insight into the ability of RNAi to modulate DENV infection, in the current study we first investigated whether S2 cells are susceptible to DENV infection. S2 cells are an attractive substrate for investigation of RNAi for three reasons: (i) the RNAi pathway in *Drosophila *is well characterized, (ii) RNAi knockdown in S2 cells can be accomplished simply be overlaying them with dsRNA or siRNA [19], and (iii) previously validated siRNA's for knockdown of specific RNAi enzymes are readily available [20,21]. After finding that DENV replicates in S2 cells, we tested whether S2 cells respond to DENV infection by production of siRNA. Finally, we tested the impact of individually knocking down four enzymes of the RNAi pathway: Dcr-1, Dcr-2, Ago-1 and Ago-2 on the replication dynamics of DENV.

## Methods

### Cells

Schneider S2 cells (*Drosophila melanogaster *embryonic cells) [22] acquired from the *Drosophila *Genomics Resource Center (Bloomington, IN) were maintained at 28°C in conditioned S2 media composed of Schneider's *Drosophila *media (Invitrogen, Carlsbad, CA) supplemented with 10% Fetal Bovine Serum (FBS, Invitrogen), 1 mM L-glutamine (Invitrogen), and 1× Penicillin-Streptomycin-Fungizone^® ^(PSF, Invitrogen). Media used for dsRNA/siRNA dilutions (unconditioned S2 media) was Schneider's *Drosophila *media supplemented with 1 mM L-glutamine and 1× PSF. C6/36 cells (*Ae. albopictus *epithelial cells) [23] were maintained at 32°C with 5% CO_2 _in minimal essential media (MEM, Invitrogen) supplemented with 10% FBS, 2 mM L-glutamine, 2 mM nonessential amino acids (Invitrogen) and 0.05 mg/ml gentamycin (Invitrogen).

### Viruses

To compare the replication of the four serotypes of DENV, three isolates of each were selected from a broad array of geographical locations (Table 1). Each isolate was passaged in C6/36 cells to generate a stock, designated C6/36 p1 MOI 0.1, for use in all experiments. C6/36 cells were infected at MOI 0.1, incubated for two hrs with occasional, gentle rocking under the conditions described above. Five days post infection (pi), supernatant was collected, clarified by centrifugation, stabilized with 0.1 times volume of 10× SPG (2.18 mM sucrose, 60 mM L-glutamic acid, 38 mM potassium phosphate [monobasic], 72 mM potassium phosphate [dibasic]), and stored at -80°C. The titer of each C6/36 p1 MOI 0.1 stock was determined via serial titration in C6/36 cells as described below.

**Table 1:** Passage history and titer (in C6/36 cells) of the 12 dengue virus strains used in this study

Serotype	Strain ID	Country of isolation	Source	Collection Year	Passage History^1^	**Titer (log**_10_ pfu/ml)	Obtained from^2^
DENV-1	JKT 85-1415	Indonesia	Human serum	1985	C6/36 p2	7.2	WRCEVA

DENV-1	1335 TVP	Sri Lanka	Human serum	1981	Inoculated mosquito-1X, C6/36 p2	7.2	WRCEVA

DENV-1	AusHT15	Australia	Human serum	1983	C6/36 p2	7.5	WRCEVA

DENV-2	Tonga/1974	Tonga	Human serum	1974	Mosquito-1X, C6/36 p5	8.0	NIAID

DENV-2	DOO-0372	Thailand	Human serum	1988	Previous history unknown, C6/36 p8	8.0	NIAID

DENV-2	NGC Proto	New Guinea	Human serum	1944	Inoculated monkey- 1X	7.5	NIAID

DENV-3	89 SriLan 1: D2783	Sri Lanka	Human serum	1989	C6/36 p2	7.6	UNC

DENV-3	89 SriLan 2: D1306	Sri Lanka	Human serum	1983	C6/36 p2	7.6	UNC

DENV-3	Sleman/78	Indonesia (Java)	Human serum	1978	Mosquito-1X, Vero p2, C6/36 p4	7.2	NIAID

DENV-4	1228 TVP	Indonesia	Human serum	1978	Mosquito p2, C6/36 p2	7.1	WRCEVA

DENV-4	779157	Taiwan	Human serum	1988	C6/36 p5	7.4	WRCEVA

DENV-4	BeH 403714	Brazil	Human serum	1982	C6/36 p3	7.2	WRCEVA

^1^cell type for passage followed by total number of passages (p) in that cell type

^2 ^WRCEVA: provided by Dr. Robert Tesh at the World Reference Center of Emerging Viruses and Arboviruses at the University of Texas at Galveston (UTMB); NIAID: provided by Dr. Stephen Whitehead, NIAID, NIH; UNC: provided by Dr. Aravinda de Silva, Department of Microbiology and Immunology, University of North Carolina.

### Quantification of virus titer

Monolayers of C6/36 cells were grown to 80% confluency in 24-well tissue culture treated plates (BD Falcon, Franklin Lakes, NJ) and infected with serial tenfold dilutions of each stock virus or cell supernatant. Plates were incubated for two hrs with intermittent gentle rocking at 32°C. Inoculated monolayers were overlaid with 0.8% methylcellulose in OptiMEM (Invitrogen) supplemented with 2% FBS, 2 mM L-glutamine and 0.05 mg/ml gentamycin. Focus forming units are referred to as "plaques" hereafter for consistency with previous literature [24-28]; plaques were detected via immunostaining as previously described [29]. DENV-1 - 4 were detected using DENV - 1 specific monoclonal antibody 15F3, DENV - 2 hyperimmune mouse ascites fluid (HMAF), DENV - 3 specific hybridoma cell supernatant, and DENV- 4 HMAF, respectively; all antibodies were the kind gift of Dr. Stephen S. Whitehead, National Institute of Allergy and Infectious Disease, National Institutes of Health, Bethesda, MD.

### Infection of S2 cells by DENV

**Figure 2:**
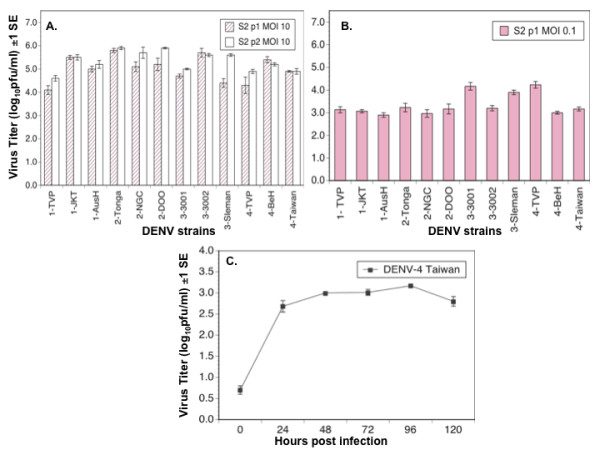
**Replication of DENV in *Drosophila melanogaster *S2 cells**. A: Titer of 12 strains of DENV 5 days post infection following passage 1 (S2 p1 MOI 10, solid bars) and passage 2 (S2 p2 MOI 10, open bars) in *Drosophila melanogaster *S2 cells. In passage 1, cells were infected with each virus strain at MOI 10. In passage 2, cells were infected with 500 μl of cell supernatant from passage 1; B: Titer of 12 strains of DENV 5 days pi following infection of S2 cells at MOI 0.1 (S2 p1 MOI 0.1); C: Replication kinetics of DENV-4 Taiwan at MOI 0.1 in *Drosophila melanogaster *S2 cells.

S2 cells were grown to 80% confluency (6.0 log_10 _cells/well ± 3.1 log_10 _cells/well) in six-well tissue culture treated plates (BD Falcon). Triplicate wells were infected with each of the 12 C6/36 p1 MOI 0.1 stocks at a specified MOI, based on titer in C6/36 cells (Table 1) divided by the number of S2 cells/well, in a total volume of one ml. Virus was incubated for two hrs at 28°C with occasional, gentle rocking and washed once with one ml of conditioned S2 media. Thereafter three ml of conditioned S2 media was added to each well. S2 cells were infected at MOI 10 and incubated for five days at 28°C after which cell supernatants, designated S2 p1 MOI 10, were collected and frozen as described above. 500 μl from each S2 p1 MOI 10 replicate were then passaged in fresh S2 cells as described above. Given the titers on day five for S2 p1 MOI 10 (Figure 2A), 500 μl of supernatants contained a total of 3.2 - 4.4 log_10_plaque forming units (log_10_pfu). Cells were incubated for five days and harvested to yield S2 p2 MOI 10. S2 cells were infected similarly at MOI 0.1 to yield cell supernatants S2 p1 MOI 0.1, but these supernatants were not passaged further. Virus titer in all cell supernatants was determined by serial titration in C6/36 cells as described above.

### DENV replication kinetics in S2 cells

Triplicate wells of S2 cells in six-well plates were infected with the C6/36 p1 MOI 0.1 stock of DENV-4 Taiwan at MOI 0.1. Two hrs post infection the inoculum was removed, cells were washed once with conditioned S2 media, fresh media was added and 1 ml cell supernatant was collected from each well 2, 24, 48, 72, 96 and 120 hrs pi and frozen as described above. Fresh media was added to each well for every sampling point so that the total volume of media remained constant.

### Detection of anti-DENV siRNAs in S2 cells

Northern blots were used to detect anti-DENV siRNAs in infected S2 cells. To assess the production of siRNA's in response to infection, one set of S2 cells at 80% confluency were infected with DENV-1 JKT, DENV-2 Tonga, DENV-3 Sleman and DENV-4 Taiwan at MOI 0.1 as described above. To assess the impact of knocking down components of the RNAi pathway on siRNA production, a second, concurrent set of S2 cells were treated with dsRNA to Dcr-1 or Dcr-2 and then infected with DENV-1 JKT, DENV-2 Tonga, DENV-3 Sleman and DENV-4 Taiwan as described below. Three days pi small RNAs (15 - 100 nucleotides) were isolated using mirPremier^® ^microRNA Isolation kit (Sigma Aldrich, St. Louis, MO). RNA was quantified, separated on 15% urea polyacrylamide gel using Tris Borate EDTA and transferred to Hybond™-N+ nylon membrane (Amersham Biosciences, Pittsburgh, PA). Blots were probed with approximately 400 nucleotide long digoxigenin (DIG) labeled positive-sense probes complementary to nucleotides 10271 - 10735 of the 3' untranslated region (UTR) of DENV-1 Western Pacific, 10270 - 10713 of the 3'UTR of DENV-2 Tonga, 10243 - 10686 of the 3'UTR of DENV-3 Sleman and 10240 - 10645 of the 3'UTR of DENV-4 Taiwan. The justification for targeting the probe to the 3' UTR is based on a recent report that anti-West Nile virus siRNA's cluster, among other genome locations, in the 3' UTR [30]. Blots were processed according to protocol defined by the manufacturer for DIG probes (Roche Diagonistics, Indianapolis, IN).

### Knockdown of enzymes in the RNAi pathway

Four components of the RNAi pathway, Ago-1, Ago-2, Dcr-1 and Dcr-2 (Figure 1) were separately depleted using 500 base-pair (bp) dsRNA targeting nucleotides 140 - 641 of Dcr-1, 763 - 1264 of Dcr-2, 1151 - 1651 of Ago-1 mRNA from *D. melanogaster *[Genbank: NM_079729, NM_079054, DQ398918 respectively] or a previously validated 22 bp siRNA against *D. melanogaster *Ago-2 [20]. A dsRNA targeting nucleotides 72 - 573 of pGEX-2T cloning vector (GE Healthcare Life Sciences, Piscataway, NJ) was used as a control for dsRNA knockdown while a *Renilla *luciferase siRNA (Ambion, Austin, TX) targeting luciferase was used as control for siRNA knockdown. To generate dsRNA, *D. melanogaster *DNA was isolated using the Qiagen DNeasy Blood & Tissue Kit (Qiagen, Valencia, CA, USA) and amplified using primers specific to *D. melanogaster *Ago-1, Ago-2, Dcr-1 and Dcr-2 (Table 2). Primers contained a T7 promoter sequence at the 5' end to allow for transcription using MEGAscript^® ^RNAi Kit (Ambion) according to manufacturer's instruction. Transcription of siRNA was performed using Silencer^® ^siRNA construction kit (Ambion). 6.0 log_10 _± 3.0 log_10 _S2 cells were plated on six-well plates and incubated for 20 minutes at 28°C. dsRNA/siRNA were diluted in one ml of unconditioned S2 media to 100 nM, applied to the S2 cells, and incubated at 28°C for 16 hrs. Thereafter three ml of conditioned S2 media was added and cells were incubated as described above [31]. Cells were re-fed with dsRNA/siRNA three days following initial treatment.

**Table 2:** Primers used for amplification of targets for dsRNA generation

Primer Name	Primer sequence^1^	Protein
Dicer-1-Forward	CTAATACGACTCACTATAGGGCGGAACACGATTATTTGCCTGGG	Dicer-1

Dicer-1 Reverse	CTAATACGACTCACTATAGGGCGCAACACGGTGACAATATCACTG	Dicer-1

Dicer-2 Forward	CTAATACGACTCACTATAGGGAAGAGCAAGTGCTCACGGTTACAAG	Dicer-2

Dicer-2 Reverse	CTAATACGACTCACTATAGGGGCGTAGACTGGATGTAGTTGAGCA	Dicer-2

Argonaute-2 Forward	CTAATACGACTCACTATAGGGCATCAACTATCTGGACCTTGACCTG	Argonaute-2

Argonaute-2 Reverse	CTAATACGACTCACTATAGGGAAACAACCTCCACGCACTGCATTG	Argonaute-2

dsRNAControl-Forward	CTAATACGACTCACTATAGGGCAGGTCGTAAATCACTGCATAATTC	Control

dsRNAControl-Reverse	CTAATACGACTCACTATAGGGCACCGTATCTAATATCCAAAACCG	Control

^1 ^5' to 3' sequence

### Verification of Knockdown

To assess the efficacy of knockdown, seven wells of S2 cells were treated with each of the dsRNA/siRNA's described above. At two hrs, 24 hrs, and daily thereafter through day six post-treatment, cells from one well corresponding to each dsRNA/siRNA treatment were lysed using RIPA buffer (Thermo Scientific, Waltham, MA) and centrifuged for 25 minutes at 10,000 rpm at 4°C. Supernatants were stored at -80°C in order to analyze all samples concurrently. Total protein in each sample was quantified using BCA Protein Assay kit (Pierce, Rockford, IL). Supernatants were separated on a polyacrylamide gel and transferred to Immobilon polyvinylidene fluoride transfer membranes (Millipore, Billerica, MA). Membranes were blocked with bovine serum albumin and incubated with *D. melanogaster *specific anti-Dcr-1 (Catalog number: ab52680), anti-Dcr-2 (Catalog number: ab4732), anti-Ago-1 (Catalog number: ab5070), or anti-Ago-2 antibody (Catalog number: ab5072) (Abcam, Cambridge, MA) as appropriate. Protein bands were visualized with secondary anti-rabbit or anti-mouse HRP-conjugated IgG (Kirkegaard and Perry Laboratories, Gaithersburg, MD) using the ECL system (GE Healthcare).

### Toxicity assay

To assess whether knockdown of Dcr-1, Dcr-2, Ago-1 or Ago-2 affected the viability of S2 cells, a resazurin-based viability assay was performed. S2 cells were propagated to 80% confluency in five 96 well tissue culture treated plates (Costar, Lowell, MA). Each treatment was performed in triplicate wells on each plate as follows. Media was removed and designated dsRNA/siRNA's were added at a concentration of 100 nM. Two controls were included in the assay: treatment with 100 μl of conditioned S2 media was used to measure overall cell viability and treatment with 8% DMSO was used to measure the impact of a compound known to be toxic. Plates were incubated for one to five days; on each day 100 μl of resazurin from the *In Vitro *Toxicology Assay Kit (Sigma-Aldrich, St. Louis, MO) was added all the wells of one plate. The plate was then incubated two hrs and absorbance was read on a plate reader (TiterTek, Huntsville, AL) at 600 nm. The proportion of viable cells was determined by dividing the absorbance of each well on the plate by the average absorbance of the media-treated wells.

### DENV infection following knockdown of Dcr-2

For each of the C6/36 p1 MOI 0.1 stocks of 12 DENV strains (Table 1), triplicate wells of S2 cells in six-well plates were treated with dsRNA targeting Dcr-2 or with control dsRNA as described above. Sixteen hrs post treatment wells were infected with the designated virus strain at MOI 10 and incubated at 28°C. Based on the results of knockdown verification (below), infected cells were replenished with dsRNA 72 hrs pi. Cell supernatants were carefully removed and stored in individual tubes at room temperature, leaving one ml residual supernatant per well. 100 nM dsRNA was added to each well and incubated for 30 minutes at 28°C. Each cell supernatant that was removed was added back to its original well containing one ml of residual media. Cell supernatants were harvested 120 hrs pi and virus titer was determined as described above.

### DENV replication kinetics following knockdown of Dcr-1, Dcr-2, Ago-1 or Ago-2

To monitor the impact of RNAi knockdown on DENV replication kinetics, sets of six wells of S2 cells in six-well plates were treated with one dsRNA/siRNA targeting Dcr-1, Dcr-2, Ago-1, Ago-2 or one control dsRNA/siRNA, as described above. 16 hrs post treatment, three wells treated with each enzyme were infected with DENV-4 Taiwan and three with DENV-2 Tonga at MOI 10. One ml cell supernatant was collected from each well 2, 24, 48, 72, 96 and 120 hrs pi and frozen as described above; one ml of fresh media was then added to each well so that the total volume of media remained constant. All wells were re-fed dsRNA/siRNA at 72 hrs pi as described above.

### Statistical Analysis

All statistical analyses were carried out using Statview (SAS Institute, Cary, NC).

## Results

### Infection of S2 cells by DENV

Every DENV strain achieved a titer > 7.0 log_10_pfu/ml in C6/36 cells five days post-infection at MOI 0.1 (Table 1). Five days after infection of S2 cells at MOI 10, the 12 DENV strains reached titers ranging from 4.1 to 5.9 log_10 _pfu/ml (Figure 2A). There was a significant positive correlation between titer of the 12 DENV strains in C6/36 (C6/36 p1 MOI 0.1) with the titer of those strains in S2 (S2 p1 MOI 10) (r = 0.62, P = 0.03). There was no significant difference among the four DENV serotypes in titer following this first passage in S2 cells (ANOVA, df = 3, F = 2.54, P = 0.13), and titer did not change significantly following a second passage in S2 cells, S2 p2 MOI 10 (Figure 2A; paired t-test, df = 11, P = 0.66).

To confirm that the titers observed in S2 cells resulted from virus replication rather than carry-over of the inoculum, S2 cells were also infected with all 12 strains of DENV at MOI 0.1; five days pi all 12 strains had achieved titers ranging from 2.9 to 4.2 log_10 _pfu/ml (Figure 2B). There was no significant correlation between titers of the 12 strains following infection of S2 cells at MOI 0.1 (S2 p1 MOI 0.1) and MOI 10 (S2 p1 MOI 10) (r = - 0.55, P = 0.06) or between titers of the 12 strains following infection of S2 cells at MOI 0.1 (S2 p1 MOI 0.1) and C6/36 cells at MOI 0.1 (C6/36 p1 MOI 0.1) (r = - 0.19, P = 0.54). Additionally the replication kinetics of one strain, DENV-4 Taiwan, were followed daily for five days (Figure 2C); there was significant difference in virus titer among days post-infection (repeated measures ANOVA, df = 5, F = 113.09, P < 0.0001); specifically, a Tukey-Kramer post-hoc test revealed that virus titer increased between two hrs and 24 hrs (P < 0.5) and leveled off thereafter at approximately 3.0 log_10_pfu/ml.

### Detection of anti-DENV siRNA in S2 cells

**Figure 3:**
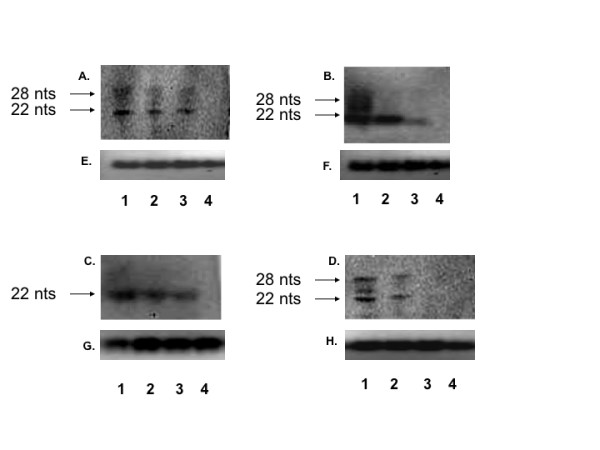
**Detection of siRNAs in S2 cells infected with specified DENV strain (Lane 1), specified DENV strain following Dcr-1 knockdown (Lane 2), specified DENV strain following Dcr-2 knockdown (Lane 3), or uninfected cells (Lane 4) by Northern blot probed with DENV 3'UTR specific probe**. A- DENV-1 TVP. B- DENV-2 Tonga. C- DENV-3 Sleman. D- DENV-4 Taiwan. E - H: Total RNA loaded for A, B, C and D, stained with ethidium bromide, as an equal loading control.

Virus-derived small RNAs can range from 18 - 30 nucleotides depending on secondary structure of the viral genome and processing by RNA processing enzymes [16,32]. Virus derived small RNAs were detected in S2 cells three days after infection with DENV-1 TVP, DENV-2 Tonga, DENV-3 Sleman and DENV-4 Taiwan by Northern blotting (Figure 3) using positive-sense probes designed to detect negative sense siRNAs that targeted the positive sense genome of each respective serotype. No virus-derived siRNA's were detected in uninfected control cells. Knockdown of Dcr-1 or Dcr-2 resulted in a substantial decrease in the production of virus-derived siRNA's in S2 cells infected with each of the four isolates above (Figure 3). The most extreme effect was apparent for Dcr-2 knockdown followed by infection with DENV-4 Taiwan; in this treatment no virus-derived siRNA's were detected at all (Figure 3D, compare lane 3 to lane 1).

### Toxicity in S2 cells following knockdown of Dcr-1, Dcr-2, Ago-1 or Ago-2

**Figure 4:**
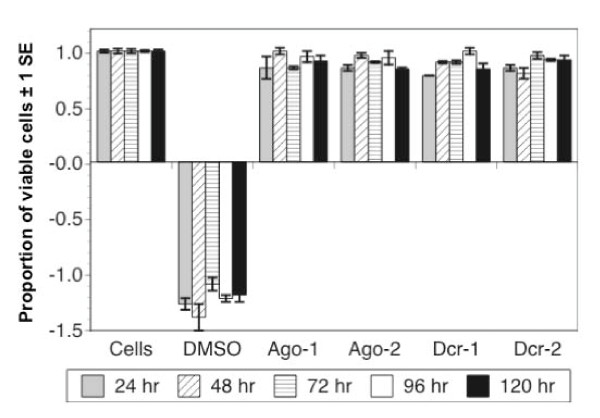
**Proportion of viable cells (absorbance of individual wells divided by mean absorbance of control wells) in cells treated with media only (cells), 8% DMSO, or dsRNA/siRNAs targeting Ago-1, Ago-2, Dcr-1 or Dcr-2**. Only DMSO significantly affected cell viability.

Knockdown of each of the four components of the RNAi pathway had no significant effect on cell viability (Figure 4). A two-factor ANOVA testing the effect of treatment and day post-infection on absorbance revealed a significant effect of treatment (df = 5, F = 88.0, P < 0.001) but not day (df = 4, F = 0.2, P = 0.91). However a Tukey-Kramer post-hoc test revealed that only the DMSO-treated cells, which were expected to show reduced viability, differed significantly from control cells (P < 0.05), while none of the dsRNA/siRNA treated cells differed from controls (P > 0.05).

### DENV replication following knockdown of RNAi genes

**Figure 5:**
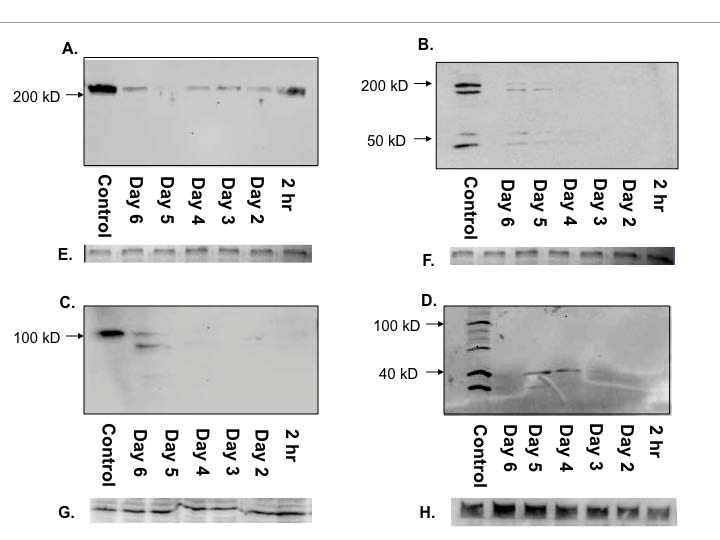
**Knock down of specific enzymes of the RNAi pathway**. Immunoblot of: A- Dcr-1 dsRNA-treated S2 cells detected with Dcr-1 antibody. B- Dcr-2 dsRNA-treated S2 cells detected with Dcr-2 antibody. C- Ago-1 dsRNA-treated S2 cells detected with Ago-1 antibody. D- Ago-2 siRNA treated-S2 cells detected with Ago-2 antibody. E - H: Actin expression for samples of A, B, C and D as an equal loading control.

To test whether the RNAi response has an effect on DENV replication in S2 cells, four components of the RNAi pathway (Dcr-1, Dcr-2, Ago-1 and Ago-2) were individually depleted via knockdown with an appropriate dsRNA or siRNA. The efficacy of depletion of each enzyme was confirmed using Western blot analysis (Figure 5). Dcr-1 levels were depleted for six days following treatment, but unlike the other three treatments there were no days on which Dcr-1 expression was undetectable. Dcr-2 expression was undetectable until day three post-treatment and showed steady recuperation thereafter. Ago-1 expression was undetectable through day five post-treatment. Ago-2 expression was undetectable until day three post-treatment and rebounded on day four. To prevent recovery of expression, all infected cell knockdowns were re-fed dsRNA/siRNA on day three post initial dsRNA/siRNA treatment.

**Figure 6:**
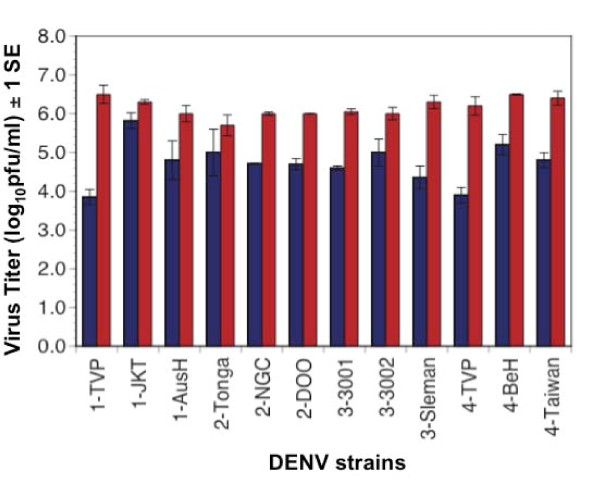
**Titer of 12 strains of DENV five days post infection in S2 cells depleted of Dcr-2 (red bars) or control cells (blue bars)**.

As shown in Figure 6, all 12 DENV strains tested achieved significantly higher titers (usually a 100-fold increase) in cells depleted of Dcr-2 relative to control cells (paired t-test, df = 11, P < 0.0001). The 12 DENV strains attained similar titers in cells treated with a control dsRNA treatment as compared to untreated cells. Moreover, there was no significant difference among serotypes in the impact of Dcr-2 knockdown, measured as the difference in titer for a particular replicate virus in knockdown cells versus control cells (ANOVA, df = 3, F = 1.04, P = 0.41). In contrast, variation in the impact of RNAi knockdown on the three DENV strains within serotypes was detected using factorial ANOVAs for each serotype; when significant differences were detected, a Tukey-Kramer post-hoc test was used to determine which strains showed significant differences in response to knockdown. DENV-1 strains showed significant variation in response to Dcr-2 knockdown (df = 3, F = 9.81, P = 0.048): strain TVP showed a significantly greater increase in titer when Dcr-2 was knocked down than strains JKT and AusH; the latter two did not differ from each other. DENV-2 and DENV-3 strains did not show significant within-serotype variation (DENV-2: df = 2, F = 2.24, P = 0.19; DENV-3 df = 2, F = 4.82 =, P = 0.06). DENV-4 strains also showed significant variation to Dcr-2 knockdown (df = 3, F = 9.8, P = 0.048): all three strains tested differed significantly from each other.

**Figure 7:**
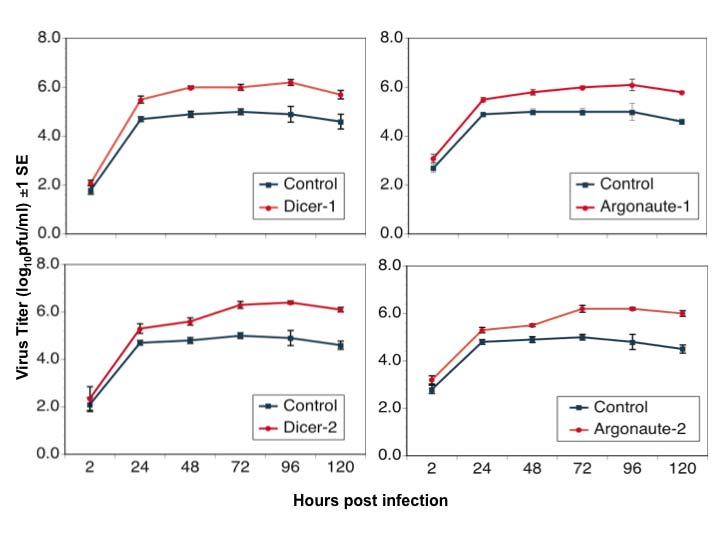
**Replication kinetics of DENV-2 Tonga in S2 cells depleted of specified components of the RNAi pathway**.

**Figure 8:**
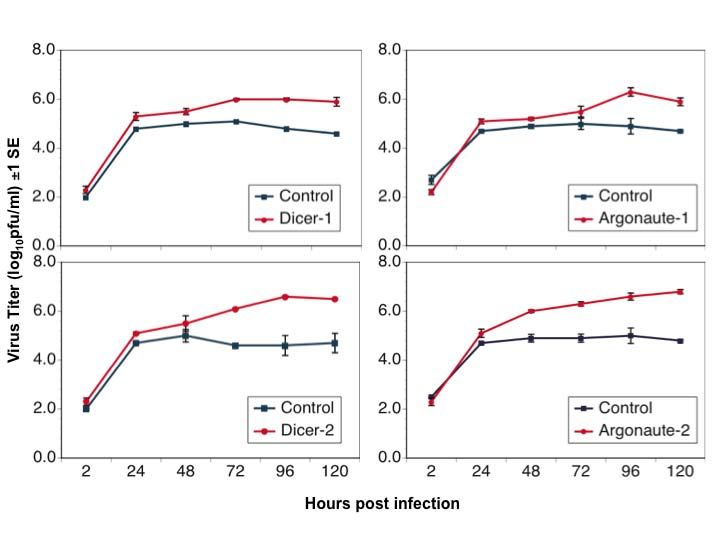
**Replication kinetics of DENV-4 Taiwan in S2 cells depleted of specified components of the RNAi pathway**.

Subsequent analyses focused on two DENV strains that had shown the smallest (DENV-2 Tonga) and an intermediate (DENV-4 Taiwan) response to Dcr-2 knockdown (Figure 6). A multistep growth curve revealed that knockdown of Dcr-2 resulted in enhancement of replication of both strains within 48 hrs pi, and by 72 hrs pi both strains had achieved a titer 10 - 100 - fold higher in Dcr-2 depleted cells than control cells (Figures 7 and 8). A similar pattern was observed following knockdown of Dcr-1, Ago-1 and Ago-2 (Figures 7 and 8); titers of both DENV strains were significantly higher in cells depleted of each enzyme than control cells 96 hrs pi (unpaired t-tests; df = 4, P < 0.02 for all comparisons).

## Discussion

The objectives of this study were threefold: first, to monitor the pattern of replication of DENV in S2 cells in order to assess the utility of S2's for the study of DENV, second to investigate the impact of RNAi on DENV replication; and third to test whether the impact of RNAi differs among the four serotypes of DENV.

Five lines of evidence demonstrate that all four DENV serotypes replicated in S2 cells. First, infection of S2 cells with DENV at an MOI 10 and MOI 0.1 resulted in titers > 4.1 and > 2.9 log_10_pfu/ml, respectively, even though the input virus inoculum was thoroughly washed away two hours post-infection. Second, titers attained by DENV following a second passage in S2 cells (4.2 - 5.9 log_10_pfu/ml) were substantially larger than the total amount of virus used to initiate infection (3.2 - 4.4 log_10_pfu). Third, daily monitoring of the titer of DENV-4 Taiwan in S2 cells showed that titers increased significantly following one day of infection. Fourth, siRNAs were detected in S2 cells after infection with each of the four serotypes of DENV, indicating that DENV infects and replicates in S2 cells. Finally, a significant increase in titer was observed for all DENV strains when Dcr-2 was knocked down using dsRNAs. Such change in titer following down regulation of an antiviral response is indicative of active replication of DENV.

There was no evidence of change in titer of DENV between a first and second passage on S2 cells. However future studies to monitor adaptation after extensive serial passage in S2 cells are planned. Sessions et al. [33] reported that DENV-2 NGC attained a peak titer of 3.0 log_10_pfu/ml in S2 derived D.Mel-2 cells without prior adaptation. Following serial passages for four months in D.Mel-2 cells, DENV-2 NGC titer increased to 5.0 log_10_pfu/ml. Consistent with these findings, in the current study peak titers of DENV in S2 cells infected at MOI 0.1 were approximately 3.0 log_10_pfu/ml [33]. However peak titers following infection at MOI 10 were at least an order of magnitude higher. Like other RNA viruses, DENV exists as a quasispecies [34-37], and it is possible that variants that were better able to infect S2 cells occurred in the larger virus population used to infect at MOI 10 (7.0 log_10_pfu) relative to MOI 0.1 (5.0 log_10_pfu). This hypothesis is supported by the finding that viruses that were taken from the MOI 10 infection and passaged again onto S2 cells achieved a similar titer to the S2 p1 MOI 10 infection, even though their founding population was only 3.2 - 4.4 log_10_pfu.

Using DENV adapted to S2 cells, Sessions et al. demonstrated the utility of these cells for investigation of dengue virus host factors (DVHF) [33]. They identified 116 DVHF using a genome-wide RNAi screen on D.Mel-2 cells. Findings from the current study indicate that S2 cells can also support replication of unadapted DENV, thereby offering additional opportunities to leverage the extraordinary depth of knowledge and plethora of tools in *Drosophila *genetics for the study of DENV [38].

The titer of each DENV strain in S2 cells was substantially lower than its titer in C6/36 cells, which are derived from *Ae. albopictus*, a natural DENV vector [39,40]. At first glance, this result seems to suggest S2 cells may not be a useful model to study DENV-vector interactions. However, it has been previously demonstrated that C6/36 cells exhibit a weak, and possibly incomplete, RNAi response [16,17], which may contribute to their ability to support high levels of DENV replication. In contrast, both live mosquitoes [41,42] and S2 cells [21,43] marshal a vigorous RNAi response to infection with flaviviruses and other RNA viruses that is capable of limiting viral replication [43-45]. Thus for some areas of study, particularly RNAi-virus interactions, S2 cells may be preferable to C6/36 cells as an *in vitro *model.

In this study S2 cells infected with DENV-1, 2, 3 or 4 produced siRNAs targeting the DENV genome, as has been reported previously for a variety of viruses, including DENV, in multiple types of insect cells both in culture and *in vivo *[41,43]. In a notable exception to this rule, C6/36 cells failed to produce siRNAs when infected with WNV [16]. The production of anti-DENV siRNA provides confirmation that DENV is targeted by an active RNAi response in S2 cells. Further, a decrease in virus derived small RNAs was observed when Dcr-2 or Dcr-1 was knocked down prior to infection. This finding supports the conclusion that enhancement of DENV replication following knockdown of components of RNAi (discussed below) resulted from a relaxation of RNAi control. Although the current study was designed to detect only siRNAs complementary to the positive sense 3' UTR, it would be very useful in the future to characterize the entire suite of siRNAs produced in response to DENV infection.

In *Drosophila*, virus derived small RNAs can be generated by Dcr-2 or Dcr-1 [11] and subsequently processed by Ago-1 or Ago-2-RISC (RNA Induced Silencing Complex) [46] (Figure 1). Knockdown of Dcr-2 enhanced the replication of each of 12 strains of DENV, and knockdown of Ago1, Ago-2 or Dcr-1 enhanced replication of the two DENV strains tested. None of the four knockdowns affected cell viability, supporting the conclusion that the observed augmentation of DENV replication was due to knockdown of the targeted enzymes rather than off-target effects. There was no difference in the impact of the four enzymes on DENV replication dynamics, and there was no difference among serotypes in their average response to the knockdown of Dcr-2. Intriguingly, strains within DENV-1 and DENV-4 serotypes showed significant variation in their response to Dcr-2 knockdown. These data suggest that DENV strains may vary in their sensitivity to RNAi, potentially contributing to differences in viral replication in the vector with downstream effects on transmission. Although the current study was not designed to draw inferences about response of specific DENV genotypes to RNAi or to contrast isolates associated with different grades of disease severity, the S2 system could be used to address these questions in the future.

The impact of Dcr-2 and Ago-2 knockdowns in this study are generally consistent with the results of Sanchez-Vargas et al. [18], who found that knockdown of either enzyme in *Ae. aegypti in vivo *enhanced replication of DENV-2, although the impact of Ago-2 knockdown was delayed in time relative to Dcr-2. However our results in S2 cells differ from the finding of Chotkowski et al. that loss of Dcr-2 expression in S2 cells did not affect WNV replication [16]. This disparity may reflect methodological differences, particularly differences in expression of RNAi-pathway proteins between S2 cell lines, or differences between WNV and DENV in sensitivity to RNAi, and/or differences between the two viruses in their tendency to elicit RNAi.

Other studies have also revealed variation among viruses in their sensitivity to loss of Dcr-2 function. *Drosophila *carrying a homozygous null mutation for Dcr-2 were hypersusceptible to infection by *Drosophila *C virus (DCV) and cricket paralysis virus [47], and loss of function of Dcr-2 in *Drosophila *also resulted in increased infection by Flock House virus, DCV and Sindbis virus [48]. In contrast, homozygous knockout of Dcr-2 in *Drosophila *had no impact on susceptibility to *Drosophila *X virus (DXV) [49]. In the studies that detected no impact of Dcr-2 function on replication of WNV or DCV, respectively [16,49], the authors suggested that synthesis of siRNA by Dcr-1 may counteract the effect of loss of Dcr-2.

In the current study, knockdown of either Dcr-1 or Ago-1 enhanced DENV replication to a degree similar to each other and to Dcr-2 and Ago-2. These findings indicate that the proteins are functionally linked between the miRNA and siRNA braches of the RNAi pathway and thus impact viral replication. These findings are consistent with the report that *Drosophila *carrying a homozygous null mutation for *Aubergine *(an Ago-1 homolog) exhibit increased susceptibility to DXV infection [49] and support the idea that Dcr-1 and Ago-1 also regulate virus replication. Such regulation likely stems from the activity of Dcr-1 and Ago-1 in the siRNA branch of the RNAi pathway. Evidence of such activity includes the requirement of Dcr-1 for mRNA degradation [11], the observation of similar transcript profiles in cells depleted of Ago-1 and Ago-2 [50], and the weak association of Ago-1 with siRNAs in cells depleted of Ago-2 [46]. From this perspective, it would be particularly interesting in future studies to assess the impact of concurrent knockdown of Dcr-1 and Dcr-2 or Ago-1 and Ago-2 on the dynamics of DENV replication.

## Conclusion

Our results indicate that RNA interference regulates DENV replication in *Drosophila *S2 cells, and that DENV strains, but not serotypes, vary in their sensitivity to such regulation. S2 cells offer a useful model for the study of DENV-RNAi interactions.

## Abbreviations

RNAi: RNA interference; DENV: dengue virus; DENV-1-4: dengue virus serotypes 1-4; WNV: West Nile virus; DCV: Drosophila C virus; DXV: Drosophila X virus; Ago: Argonaute; Dcr: Dicer; siRNA: short interfering RNA; miRNA: microRNA; Spn: Spindle; pi: Post Infection; DIG: digoxigenin; UTR: untranslated region; bp: base pair; siRISC: RNA Induced Silencing Complex associated with siRNA; RISC: RNA Induced silencing complex; miRNP: miRNA associated Ribo Nucleoprotein Complex; miRISC: miRNA associated RISC; dsRNA: double stranded RNA; MOI: multiplicity of infection; PSF: Penicillin-Streptomycin-Fungizone^®^; MEM: Minimum Essential Media; hr: hour; df: degrees of freedom; p1: passage 1; p2: passage 2.

## Competing interests

The authors declare that they have no competing interests.

## Authors' contributions

Experiments were conceived by SM and KAH and performed by SM. Data was analyzed by SM and KAH. The manuscript was written by SM and KAH. All authors have read and approved the final manuscript.
